# Flock Factors Correlated with Elevated Mortality in Non-Beak Trimmed Aviary-Housed Layers

**DOI:** 10.3390/ani12243577

**Published:** 2022-12-17

**Authors:** Käthe Elise Kittelsen, Fernanda Tahamtani, Randi Oppermann Moe, Pall Gretarsson, Guro Vasdal

**Affiliations:** 1 Animalia-The Norwegian Meat and Poultry Research Centre, N-0513 Oslo, Norway; 2 Faculty of Veterinary Medicine, NMBU—Norwegian University of Life Sciences, N-1433 Ås, Norway

**Keywords:** layers, non-beak trimmed, animal welfare, mortality, aviary, non-cage housing, plumage condition

## Abstract

**Simple Summary:**

There is increasing use of non-cage housing systems for laying hens in Europe. In Norway, approximately 85% of all hens are housed in aviaries. Such systems are more complex and house larger animal groups. Knowledge of flock level factors that may affect mortality in these systems is important to be able to improve animal welfare, reduce mortality and enhance sustainability. The aim of this study was to investigate factors that may contribute to mortality in non-beak trimmed aviary-housed laying hens in Norway. Overall, the investigations found an association between elevated mortality and increased feather loss, which may be an indication of feather pecking and cannibalism.

**Abstract:**

The use of non-cage housing systems for layers is increasing in Europe and elsewhere. Knowledge of factors that may affect mortality in these systems is important to be able to improve animal welfare, reduce mortality and enhance sustainability. The aim of this study was to investigate factors that may contribute to increased mortality in non-beak trimmed aviary-housed laying hens in Norway. A total of 39 non-beak trimmed commercial flocks (Lohmann LSL (*n* = 25) and Dekalb White (*n* = 14)) were visited between week 70 to 76 of life, and factors related to health, behaviour and management were recorded. Mean mortality in the flocks was 3% (range: 0.5–9%) and increased flock mortality was correlated with total feather loss (*p* < 0.05); feather loss on the breast (*p* < 0.02) and feather loss on the head (*p* < 0.003). There was an association between layer hybrid line and mortality (*p* = 0.055). Furthermore, a low positive correlation between mortality and dust level inside the barn was found (*p* < 0.04), showing that mortality was higher when dust level was also high. No correlation between mortality and the provision of environmental enrichment was found. In conclusion, this study found an association between flocks with elevated mortality (>3.0%) and increased feather loss which may indicate feather pecking. The results underline the importance of regularly assessment of plumage condition in commercial layer farms, as a tool to detect early signs of feather pecking in commercial aviary-housed layer flocks. This may help to target feather pecking before cannibalism breaks out.

## 1. Introduction

Due to animal welfare concerns, the European Union implemented a ban on conventional battery cages for laying hens in 2012 [1]. Today, legal housing systems within the EU include enriched cages, floor, aviary, free-range, organic or mobile housing. Aviaries are the most prevalent housing systems in Northern Europe, and their use is increasing elsewhere [2,3]. In Norway, approximately 93% of all eggs originate from aviaries, free-range farms or organic farms [4]. 

Aviary systems give the hens more opportunity for natural behaviour and freedom of movement [5,6], especially if the systems are designed to promote species-specific behaviour, such as the addition of ramps. However, these systems are also associated with higher mortality rates [7,8,9,10]. Mortality rate is an important measure of animal health and welfare and as such, aviary housing may pose a threat to the welfare of the birds. In 2019, the mortality in enriched cages in Norway, where no birds are beak trimmed according to Norwegian legislation [11], was 1.96% versus 3.44% in aviary-housed layers [4]. Regardless of housing, this is a low number compared to previously reported numbers from other countries, which can range from 5.4 to 11.8% [12,13,14] and extreme cases showing mortality as high as 18% in non-beak trimmed flocks [15]. The combination of increased use of aviary housing along with an elevated mortality in these production systems, makes it imperative to investigate the factors contributing to this mortality. 

In general, several factors may affect mortality in layers, e.g., diseases, abnormal behaviours and suboptimal environment, such as poor air quality. Common diseases that may cause mortality in commercial layers are *Escherichia coli* infections, egg yolk peritonitis, gout, salpingitis, septicaemia, fatty liver haemorrhagic syndrome and prolapsed vent [8,14,16,17].

One of the most common abnormal behaviours and welfare concerns in aviary-housed laying hens is feather pecking and cannibalism [5,18]. Severe feather pecking has been estimated to occur in 40 to 50% of loose-housed layer flocks in Europe [13,19] and it may be associated with the genetics of the birds [20]. Feather pecking increases the risk of cannibalism [21], which is defined as death from tissue trauma and haemorrhage inflicted by conspecifics [16]. It has been suggested that feather pecking may indicate unfulfilled needs and hence plumage condition may serve as an indicator of reduced welfare [7]. Poor plumage condition may be an early sign of severe feather pecking [22]. Furthermore, feather pecking and cannibalism is associated with increased mortality [16,18,23] Therefore, it is important to regularly evaluate plumage condition in all layer flocks, as an early warning of feather pecking, reduced welfare and increased mortality risk. This is particularly important in aviary-housed flocks, since feather pecking is more difficult to identify in large aviary-housed animal groups versus small animal groups housed in cages [23]. Feather pecking is largely accepted as redirected ground pecking due to an unsatisfied behavioural need for foraging or dustbathing [24]. Therefore, providing pecking substrates and other environmental enrichment is a management strategy that aims at increasing animal welfare by meeting the birds’ behavioural needs, and reducing the incidence of behavioural problems [25]. 

European Union legislation requires the farmers to provide dust bathing material for laying hens [1]. While this provision meets behavioural needs, it can negatively affect the aerial environment and potentially the health of the birds. Suboptimal aerial environment with dust and high levels of CO_2_ and ammonia may impose a threat to health and welfare of layers [26], especially in light of the unique avian respiratory system [27]. Loose housing systems have been found to have higher concentrations of dust than cage systems [26], which may be due to lack of litter and dust bathing material in cage systems. Another cause may be an increased activity and higher litter build up during the production period in non-cage systems. Airborne microorganisms can be attached to dust particles, and dust may as such function as a vector for pathogens, in addition to making the birds more susceptible to infections by irritating their complex avian respiratory system [26]. Furthermore, ammonia is an aversive gas for poultry and high concentrations of gaseous ammonia can have detrimental health effects with lesions in the respiratory tract, in addition to predispose the birds to secondary infections, irritation or mortality [28]. Therefore, it is imperative to investigate how the aerial environment in fully enclosed houses affects mortality in aviary-housed laying hens. 

In order to improve animal health and welfare for aviary-housed laying hens, the objective of this study was to gain more knowledge of factors affecting cumulative mortality rates in Norwegian laying hen flocks, measured at the end of lay. To identify risk factors for mortality, the study used clinical observation of the flocks, including plumage condition, along with measures of air quality and the provision of environmental enrichment. 

## 2. Materials and Methods

This study was conducted between April 2020 and June 2021. A total of 39 commercial, non-beak-trimmed flocks (1 flock/farm) were included, summing up to a total of 307,944 laying hens. All birds were of white layer hybrids, either Lohmann LSL (*n* = 25) or Dekalb White (*n* = 14), the most commonly used layer hybrid lines in Norway. The studied flocks were randomly selected from the supplier lists of two different egg packing companies. All flocks were housed in fully enclosed, mechanically ventilated houses with multi-tiered aviary-systems, with a maximum stocking density of 9 birds per m^2^. The aviary systems had 3 tiers above the floor, feed, and water lines on tiers 1 and 2, nest boxes on tier 2, and perches on tier 3. The flocks were fed pelleted feed three times per day via a chain dispersal system, and water was provided ad libitum via drinking nipples. The hens were housed under a 14 h light/10 h dark schedule. The farmers had set temperature and humidity according to breeder manuals, approximately 20 degrees; however, these data were not controlled by the researchers. The houses were about 12 m wide, with wood shavings litter covering a floor area ranging from 385 m^2^ to 1000 m^2^ that extended around and under the tiered aviary structures. 

The flocks were visited once, between week 70 and 76. Each flock was visited in the morning, approximately 10 a.m. for all farms, by one of three researchers experienced in poultry. To avoid inter observer variability the first four flocks were visited by all three observers to assure equal scoring. During the visit, the observer collected data on mortality rates, hen weight (measured by automatic weights in the barn), feed intake (measured automatically by the barn computer), flock size, red mite infestations, and the environment in the hen room, including provision of environmental enrichment, light intensity (lux) in the hen house, and air and litter quality. The farmers’ provision of the following five types of enrichment were registered during the farm visit: gravel, grains scattered in the litter, pecking stones, oyster shells, and “toys”. The most common examples of “toy” were cut-up pieces of manure belt, plastic balls and pieces of cardboard boxes. Lux was measured at hen height with a luxometer (Extech LED meter LT40, FLIR Commercial Systems Inc., Nashua, NH, USA), in the middle row and middle section of the hen house, and in the middle of the aviary. Colour and light intensity, along with type of light were not recorded. CO_2_, NH_3_ and dust was used to evaluate the air quality. CO_2_ was measured with a CO_2_ Meter (Extech Inbstruments, CO240, Nashua, NH, USA), NH_3_ was measured by Dräger Pac 8000 (© Drägerwerk AG & Co., Lübeck, Germany). To evaluate dust, a black cardboard paper was attached to the aviary when the observer first entered the hen house. At the end of the farm visit, the cardboard was evaluated by a touch test to see how much dust had accumulated during the visit, scored on a three-point scale where 0 = low amounts of dust, 1 = intermediate dust level and 2 = high amounts of dust (Welfare Quality^®^, http://www.welfarequalitynetwork.net/en-us/reports/assessment-protocols, assecedd on 1 April 2020). Litter quality was measured in terms of moisture in the litter, also evaluated in a three-point scale where 0 = dry litter and 2 = very wet litter. Finally, the depth of the litter was measured in centimetres. 

In addition, clinical scoring of 50 randomly selected hens was recorded when walking through the barn, according to the method described in detail by Vasdal et al. [29]. The sampling spots were predetermined, so that birds from all heights and all transects were included. At the sampling spot, the second bird from the one that the assessor looked at was selected and scored for a set of welfare indicators (Table 1). Plumage condition was scored for head, back/wings, breast and tail in a 3-point scale (0–2). On the scale, 0 = no feather loss, 1 = feather loss 1–5 cm in diameter, 2 = feather loss above 5 cm in diameter. Scoring was performed visually as from approximately 0.5 to 1 m, without any handling of the birds, to minimize stress and disturbance of the flock.

Because this on-farm study did not involve any animal handling, experimental manipulations, or invasive procedures, it was exempt from approval of animal use by the Norwegian Food Safety Authority (Norwegian Regulations on Use of Animals in Research, 2015).

Statistical analyses were performed using the software SAS 9.4. The relationships between mortality, the environmental factors and clinical observation of the birds were assessed using Pearson correlations. The environmental factors were feed intake, ammonia concentration, light intensity, CO_2_ concentration, dust level score, the number of types of environmental enrichment used, litter quality score and litter depth. The results from the clinical observation were the flock level plumage score for the head, back/wings, breast, tail and a full body (sum) score, and the numbers of birds with wounds on the back and the feet. The results are presented as Pearson correlation coefficient and associated *p* values (α = 0.05). The prevalence of dirty plumage was negligible and, therefore, this parameter was not statistically analysed. The genotype of the flocks (Lohmann LSL or Dekalb White) was included in the models as a random factor. The relationship between mortality and genotype was analysed using the Glimmix procedure with a binary distribution and logit link function. A mortality score of 1 was given to flocks with mortality <3.0% and a score of 2 was given to flocks with mortality ≥3.0%.

## 3. Results

### 3.1. Mortality

**Figure 1:**
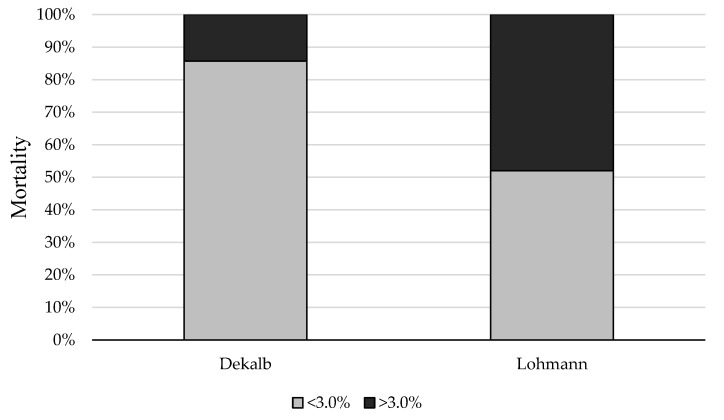
Percentage of Dekalb white (*n* = 14) and Lohman LSL (*n* = 25) flocks with mortality <3.0% and with mortality >3.0%.

Cumulative mortality rate for the 39 flocks on the day of visit ranged from 0.5 to 9.0%, with a mean of 3.0% (Table 2). There was an association between layer hybrid line and mortality (F_1,37_ = 3.94; P = 0.055), where Dekalb white flocks were more likely to have mortality < 3.0% compared to Lohmann flocks (odds ratio = 5.54). Out of the 39 flocks, 14 of them had mortality above the mean mortality rate in the study (35.9%). A total of 12 out of these 14 flocks where Lohmann (85.7%) and 2 were Dekalb (14.3%), (Figure 1).

### 3.2. Correlation between Mortality Rate and Environment Factors

Production data are presented in Table 2. Of the 39 flocks, 29 received a dust score of 0, 7 got a score 1 and 3 flocks received the maximum score of 2. Nevertheless, there was a low positive correlation between dust score and mortality (Table 3), showing that mortality was higher when the dust level was also high (*p* < 0.04). No other correlations were found between mortality rates and aerial environmental factors in the hen room, including the variables ammonia and CO_2_ concentration and litter condition. Likewise, no correlations were found between mortality rates and hen weight or feed intake. Finally, no parameter related to the use of environmental enrichment were correlated with mortality rates (Table 3). Only two flocks (4.44%) had red mite infestations. 

### 3.3. Correlation between Mortality Rate and Clinical Observation of the Birds 

Mean flock score for total feather loss was 1.8 (Table 3) indicating a relatively poor plumage condition in the flocks. Flocks with a higher plumage damage score had a higher mortality (*p* < 0.05, Table 3). In addition, increased mortality was associated with increased plumage damage score for the breast (*p* < 0.02) and head (*p* < 0.003), but not for the back and tail. The prevalence of wounds was low: 3 hens had head wounds, 3 hens had wounds to the back, 2 to the tail and cloaca and 12 to the toes. There was a weak tendency for a correlation with toe wounds and elevated mortality on flock level (*p* < 0.09, Table 3).

## 4. Discussion

This is the first systematic study to investigate factors on flock level correlated with mortality in aviary-housed laying hens in Norway. The study examined variables related to air quality, use of environmental enrichment, clinical observation of the birds, including plumage damage score and the correlations with mortality rates on flock level.

Overall, the mean mortality rates for the 39 aviary-housed flocks was 3.0%. This is relatively low compared to numbers reported in other studies, between 5.4 and 11.8% [12,13,14]. One possible explanation for the overall low mortality may be the small flock sizes. The mean flock size for laying hens in Norway is 7500, as regulated by governmental legislation [30]. This can make it easy for the farmers to observe the majority of the birds during their daily routine inspections when walking through the barn and to detect early sings of disease or suboptimal environment. In addition, there is a strong emphasis on biosecurity in Norwegian farms, making outbreaks of severe infectious diseases uncommon [4].

There was a correlation between flocks with increased mortality and increased feather loss. In addition, mortality was also associated with poor plumage on the breast and head, but not on the back and tail. Poor plumage condition may be a sign of severe feather pecking [22] which can lead to cannibalism and, in turn, increased mortality [16,18,23]. Therefore, it could be speculated that the association between poor plumage condition and elevated mortality in the current study was due to feather pecking and cannibalism. This is in line with previous research showing that flocks with better feather cover have lower levels of mortality and a positive correlation between general feather damage and mortality due to cannibalism [21,23]. Indeed, several studies have shown cannibalism to be a dominating cause of mortality in laying hens [12], especially in non-beak trimmed hens [31]. Beak trimming has been found to lower mortality and better plumage condition [32] but has not been performed in commercial Norwegian laying hens since 1974.

Even though the results may indicate feather pecking and cannibalism, no hens with severe pecking injuries were seen during the transect walks. This can have several explanations, one being the small flock sizes and management strategies adopted by the farmers. The farmers walk a minimum of two rounds in the barn each day, one of them in the morning. During these rounds, sick or dead birds are collected and dispensed. This is standard procedure and was conducted at least twice every day also prior to the researchers’ transect walk. Another explanation may be that sick or injured birds hide in the nest boxes and can be difficult to spot, resulting in an underestimation of the number of birds with pecking injuries. There was a weak tendency for a correlation with toe wounds and elevated mortality on flock level. Toe pecking can be regarded as a type of cannibalistic pecking behaviour [33]. The resulting toe injuries act as a trigger for more toe pecking, spreading the problem in the flock. Toe pecking has a low incidence and occurs sporadically, making it difficult to study [33]. Therefore, there is little available information about the effect of toe pecking on mortality, however it is linked with physiological stress and hence reduced welfare [33]. Toe pecking can be attributed to cannibalism, and complications may be lethal [34].

This study also found an association between mortality and genotype. This may be caused by feather pecking and concurring cannibalism, since different strains differ in the tendency to feather peck [20,35]. For instance, Walser (1997) studied two white layer hybrids kept under identical housing conditions and found a significant difference in the feather pecking behaviour [36]. Other causes for the association between mortality and genotype could be different susceptibility to diseases and infections [37,38]. This could not be ruled out in the current study.

The provision of environmental enrichment can reduce the incidence of feather pecking and aggressive pecking. Additionally, it improves plumage condition during both the rearing and laying period [25,31,36]. Especially the provision of litter to adult laying hens has been found to reduce the incidence of severe feather pecking, improve plumage condition and reduce mortality [39,40,41,42,43,44,45,46] and providing gravel and toys at early age is associated with reduced damage to the tail feathers [47]. No correlation between mortality and environmental enrichment was found in the current study, but a correlation between feather damage and environmental enrichment was found. This indicates that environmental enrichment may have a positive effect on feather pecking; however, it is not strong enough to counteract cannibalism and mortality. Feather pecking and foraging behaviour are related, and early access to litter substrate may influence both behaviours [48]. Information regarding rearing facility was unfortunately not available in the current study.

Air quality was measured as CO_2_ concentration, ammonia levels and dust. No correlations were found between mortality rates and CO_2_ or ammonia. This may be due to the overall good air quality in the study population; the mean CO_2_ concentration was 1590 ppm, for ammonia the mean concentration was 6.2 ppm. Three of the 39 flocks had ammonia levels above the Norwegian legal recommendations of 20 ppm, and two of the flocks had CO_2_ levels above the legal recommendation of 3000 ppm [11]. Concentrations of ammonia have been found to be higher in loose-house systems in which manure is not regularly removed, compared to cage systems [28]. One possible explanation for the relatively low levels of both ammonia and CO_2_ may be due the sampling time; the majority of the farm visits were performed during spring, summer or autumn. The reason for this was a lockdown period due to COVID-19 during the winter months of 2021. It could be speculated that the levels of ammonia would be higher if more of the farm visits were performed in the cold months, since ammonia levels are found to increase during the cold season when ventilation flow is often reduced [28]. Dust was measured with a touch-test on a black cardboard. This is not a very sophisticated method, but a practical, non-expensive and sure way to evaluate the dust level in the hen house. Loose housing systems have been found to have higher concentrations of dust than cage systems [26]. This may be due to more activity and more litter build up during the production in non-cage systems. Of the 39 flocks, 31 received a dust score of 0, 11 got a score 1 and three flocks received the maximum score of 2. Still, a low positive correlation between dust score and mortality was found, showing that mortality was higher when the dust level was also high. A dusty environment may impose a threat to health and welfare of layers [26], especially in light of the unique avian respiratory system [27]. Airborne microorganisms can be attached to dust particles, and dust may as such function as a vector for pathogens, in addition to making the birds more susceptible to infections by irritating the complex respiratory system of the birds [26], which may explain the correlation between dust and mortality found in this study.

Bacteriology was not performed; therefore, it cannot be ruled out that the increased mortality in several of the flocks was related to infectious diseases. The pathological findings in dead birds form the 39 flocks are reported in a newly published study [49]. These results indicated that keel bone fractures, fatty liver and salpingitis were the most prevalent post-mortem findings. Other causes, such as nutritional shortcomings and stress may also cause feather loss, these factors cannot be ruled out in this study. Severe feather pecking is still a serious welfare problem in aviary-housed laying hens; it is painful for the recipient [50] and it may indicate unfulfilled needs in the pecker [51], in addition to its association with increased flock mortality [52].

## 5. Conclusions

This study showed an association between flocks with increased mortality and increased feather loss. There was also a weak tendency for a correlation with toe wounds and elevated mortality on flock level. Both feather loss and toe wounds indicate feather pecking. Therefore, the results underline the importance of regular assessment of plumage condition in commercial layer farms, as a tool to detect early signs of feather pecking in aviary-housed layer flocks. This may help to target feather pecking before cannibalism breaks out.

## Figures and Tables

**Table 1:** Description of the 12 welfare indicators assessed during the clinical observation ^1^ of 50 birds.

Indicator ^2^	Description
FL ^2^ head	Missing feathers on the head, including the neck, ≥5 cm in diameter
FL back	Missing feathers on ≥50% of the back, including the wings
FL breast	Missing feathers on the breast, ≥5 cm in diameter
FL tail	Missing or clearly damaged feathers on the tail, mainly shafts and rachises left
Dirty	Prominent dark staining of the back, wing, or tail feathers, covering at least 25% of the body; not including light discoloration of feathers from dust.
Wounds head	Prominent marks on the head and neck, due to fresh or older wounds.
Wounds back	Prominent marks on the back, including the wings, due to fresh or older wounds.
Wounds tail	Prominent marks on the tail due to fresh or older wounds.
Wounds feet	Includes bumblefoot (visible dorsally), and prominent marks on the feet due to fresh or older wounds

^1^ The birds were assessed visually from 0.5–1 m distance; ^2^ Hens could be classified as belonging to more than one category; Abbreviation: FL, feather loss.

**Table 2:** Descriptive statistics of production data collected in the flocks included in the study.

Variable	Mean	Min	Max
Flock size	7896	5300	19,004
Hen weight, flock level	1775	1602	1976
Mortality rate, in %	3.0	0.5	9.0
Feed intake, flock level in grams per day	118	105	143
CO_2_, in ppm	1590	751	3800
NH_3_, in ppm	6.2	0.2	28.0
Lux ^1^,	4.3	0.9	15.0
Litter, amount in centimetres	3.5	0.5	10.0

^1^ Measured in the middle of the aviary.

**Table 3:** The relationship between mortality, environmental parameters and clinical observations.

		Descriptive Statistics				Pearson Correlation		
Variable	Unit	Mean	Std Dev	Minimum	Maximum	Coefficient	*p*-Value	N
Hen weight	g ^1^	1775	106.5	1602	1976	− 0.19	0.32	29
Feed intake	g/hen ^2^	117.62	8.54	105.00	138.00	0.17	0.30	38
NH_3_	ppm ^3^	8.46	11.51	0.00	57.00	− 0.06	0.71	39
Light intensity	lux	4.66	2.76	0.78	11.39	− 0.27	0.10	39
CO_2_	ppm	1650.00	994.05	766.67	5472.00	− 0.24	0.18	32
Dust	Score 0–2	0.33	0.56	0	2	0.33	0.04	39
Number of EEs	Type of EEs ^4^	4.27	0.94	1.00	5.00	0.05	0.76	39
Litter score	Flock average score	0.12	0.33	0.00	1.33	− 0.07	0.68	39
Litter depth	cm	3.91	2.87	0.75	13.33	− 0.19	0.25	38
FL full body	Sum of body scores	1.80	1.52	0.12	6.20	0.32	0.05	39
FL head	Flock average score 0–2	0.36	0.38	0.00	1.38	0.46	0.003	39
FL back/wings	Flock average score 0–2	0.58	0.50	0.00	1.71	0.21	0.20	39
FL breast	Flock average score 0–2	0.48	0.47	0.00	1.70	0.37	0.02	39
FL tail	Flock average score 0–2	0.40	0.47	0.00	1.48	0.02	0.91	36
Wounds back	Average number	0.14	0.27	0.00	1.36	0.05	0.76	38
Wounds feet	Average number	0.07	0.23	0.00	1.00	0.27	0.09	38

^1^ gram; ^2^ gram per hen; ^3^ parts per millimetre; ^4^ Environmental enrichment.

## Data Availability

Not applicable.
